# Blood signatures for second stage human African trypanosomiasis: a transcriptomic approach

**DOI:** 10.1186/s12920-020-0666-5

**Published:** 2020-01-30

**Authors:** Julius Mulindwa, Enock Matovu, John Enyaru, Christine Clayton

**Affiliations:** 1 Department of Biochemistry and Sports Sciences, College of Natural Sciences, Makerere University, P. O. Box 7062, Kampala, Uganda; 2 Department of Biotechnical and Diagnostic Sciences, College of Veterinary Medicine, Animal Resources and Biosecurity, Makerere University, P. O. Box 7062, Kampala, Uganda; 3 Centre for Molecular Biology of Heidelberg University (ZMBH), DKFZ-ZMBH Alliance, Im Neuenheimer Feld 282, 69120 Heidelberg, Germany

**Keywords:** Human host transcriptome, *T. b rhodesiense*, Human African Trypanosomiasis, Blood, CSF

## Abstract

**Background:**

Rhodesiense sleeping sickness is caused by infection with *T. b rhodesiense* parasites resulting in an acute disease that is fatal if not treated in time. The aim of this study was to understand the global impact of active *T. b rhodesiense* infection on the patient’s immune response in the early and late stages of the disease.

**Methods:**

RNASeq was carried out on blood and cerebral spinal fluid (CSF) samples obtained from *T. b. rhodesiense* infected patients. The control samples used were from healthy individuals in the same foci. The Illumina sequenced reads were analysed using the Tuxedo suite pipeline (Tophat, Cufflinks, Cuffmerge, Cuffdiff) and differential expression analysis carried out using the R package DESeq2. The gene enrichment and function annotation analysis were done using the ToppCluster, DAVID and InnateDB algorithms.

**Results:**

We previously described the transcriptomes of *T. b rhodesiense* from infected early stage blood (*n* = 3) and late stage CSF (*n* = 3) samples from Eastern Uganda. We here identify human transcripts that were differentially expressed (padj < 0.05) in the early stage blood versus healthy controls (*n* = 3) and early stage blood versus late stage CSF. Differential expression in infected blood showed an enrichment of innate immune response genes whereas that of the CSF showed enrichment for anti-inflammatory and neuro-degeneration signalling pathways. We also identified genes (C1QC, MARCO, IGHD3–10) that were up-regulated (log_2_ FC > 2.5) in both the blood and CSF.

**Conclusion:**

The data yields insights into the host’s response to *T. b rhodesiense* parasites in the blood and central nervous system. We identified key pathways and signalling molecules for the predominant innate immune response in the early stage infection; and anti-inflammatory and neuro-degeneration pathways associated with sleep disorders in second stage infection. We further identified potential blood biomarkers that can be used for diagnosis of late stage disease without the need for lumbar puncture.

## Background

Human African trypanosomiasis (HAT) is a neglected tropical disease that is endemic in sub Saharan Africa countries with an estimated 65 million people at risk. The disease mainly affects remote rural communities but, with the continued control activities, the number of cases reported has dropped below 10,000 (WHO fact sheet, 2018). HAT is caused by two distinct subspecies of the African trypanosomes transmitted by tsetse flies (*Glossina* spp); *Trypanosoma brucei rhodesiense* causes the acute Rhodesiense form of the disease in east and southern Africa, while in central and west Africa. *T*. *b gambiense* causes the chronic Gambiense form of the disease [1]. Uganda is the only country that has foci of both diseases [2]. The disease is characterized by two main clinical stages; an early hemolymphatic stage and a late meningoencephalitic stage where the trypanosomes cross the blood–brain barrier into the central nervous system (CNS). This encephalitic stage involves sensory, motor and psychiatric disturbances, with alterations of sleep representing the most typical manifestation [3, 4]. The only available means of screening active *T.b rhodesiense* infections is microscopy on thin films of peripheral blood (early stage) of cerebral spinal fluid obtained via lumber puncture (late stage) [5].

During the early phase of trypanosome infection in the mammalian host, there is activation of the innate immune system, which triggers B- and T-cell responses to parasite antigens, predominantly the variable surface glycoprotein (VSG). This results in Th1 pro-inflammatory cytokine profile that includes TNF-α, IL-6 and NO production [6]. Through antigenic variation, the parasites are able to evade the immune system and modify its effector function and thus sustain infection by remaining in circulation [7]. The second phase of CNS invasion activates chemokines which promote macrophage and lymphocyte recruitment to areas where their activity might induce additional alterations [8, 9]. A number of studies have been carried to understand the mechanisms of trypanosome infections and invasion of CNS, however most of these have been done in animal and in vitro blood brain barrier models [10–13]. In human *T. b rhodesiense* infections, immune responses have been observed through antibody assays and protein measurements [14–16]. The limitation to this is that only stable highly abundant molecules can be measured, leaving the low and transiently expressed proteins un-captured. A transcriptome approach could be more sensitive by measuring the RNA transcripts that could possibly explain the expressed proteins and thus pathways involved in the immune response.

We previously described the transcriptomes of trypanosomes from the blood and cerebrospinal fluid (CSF) of *T. b rhodesiense*-infected sleeping sickness patients from Eastern Uganda [17]. The sequenced samples included not only trypanosomes, but also the human cellular components. Here, we use the same dataset in order to compare gene expression in the human cells from early and late stage sleeping sickness patients. The results provide insights into immune activation and also identification of potential blood markers for late-stage disease.

## Methods

### Study site, sample collection, and RNA sequencing

Patient selection, RNA preparation and sequencing were described previously [17]. Briefly, samples were obtained from parasitologically diagnosed patients in the course of routine diagnosis. CSF was taken only from confirmed cases. Since we also wanted trypanosome transcriptomes, we selected the samples with highest parasitaemia. Blood samples were placed directly into Paxgene tubes whereas CSF samples were centrifuged and the cells resuspended in Trizol. RNA samples were prepared for sequencing using the Illumina TruSeq Total Stranded RNA preparation kit (Illumina, RS-122-2301) and sequenced using the Illumina NextSeq500 System at the EMBL Genomics Core Facility, giving 75 nt Single End reads. The raw data are available at Array express under accession numbers E-MTAB-5293 and E-MTAB-5294.

### RNA-Seq read counting and differential expression analysis

RNAseq datasets were retrieved as FASTQ files containing single end read data. The sample files were checked for quality using FastQC [18], and processed for transcript read counting following the RNASeq pipeline [19]. Briefly, alignment of reads to the Human genome GRCh38 build using Tophat, assembly of the transcripts was carried out using Cufflinks guided by the reference annotation Homo_sapiens.GRCh38.86.gtf, merging the separately assembled transcripts into one cohesive set using Cuffmerge and identifying the differentially expressed transcripts using Cuffdiff. The Cuffdiff output of gene counts was then analyzed for differential gene expression using the R package CummeRbund [20] and also based on negative binomial distribution using R package DESeq2 [21]. The DESeq2 analysis was carried out separately for differential analysis between a): Cases and Controls for paxgene blood extracted RNA and b); Cases-blood (paxgene blood) and Cases-CSF (Trizol CSF cells). Genes with counts less than 10 were filtered out. Genes which pass an adjusted *p*-value (padj) < 0.1 were considered significant. Gene annotation was carried out using the gene.gtf file from Ensembl (https://www.ensembl.org/info/website/upload/gff.html). Data transformation was carried out using the ntd, vsd and rld algorithms for which the most appropriate was selected for kmean clustering on the heatmaps. Gene functional and enrichment analysis was carried out by using the significant differentially expressed genes as queries for tools such as ToppCluster [22], the Database for Annotation, Visualization and Intergrated Discovery (DAVID) [23], the innate immune response pathway analysis InnateDB [24] and network analysis using XGR software [25].

## Results

### Study sample characteristics

**Fig. 1:**
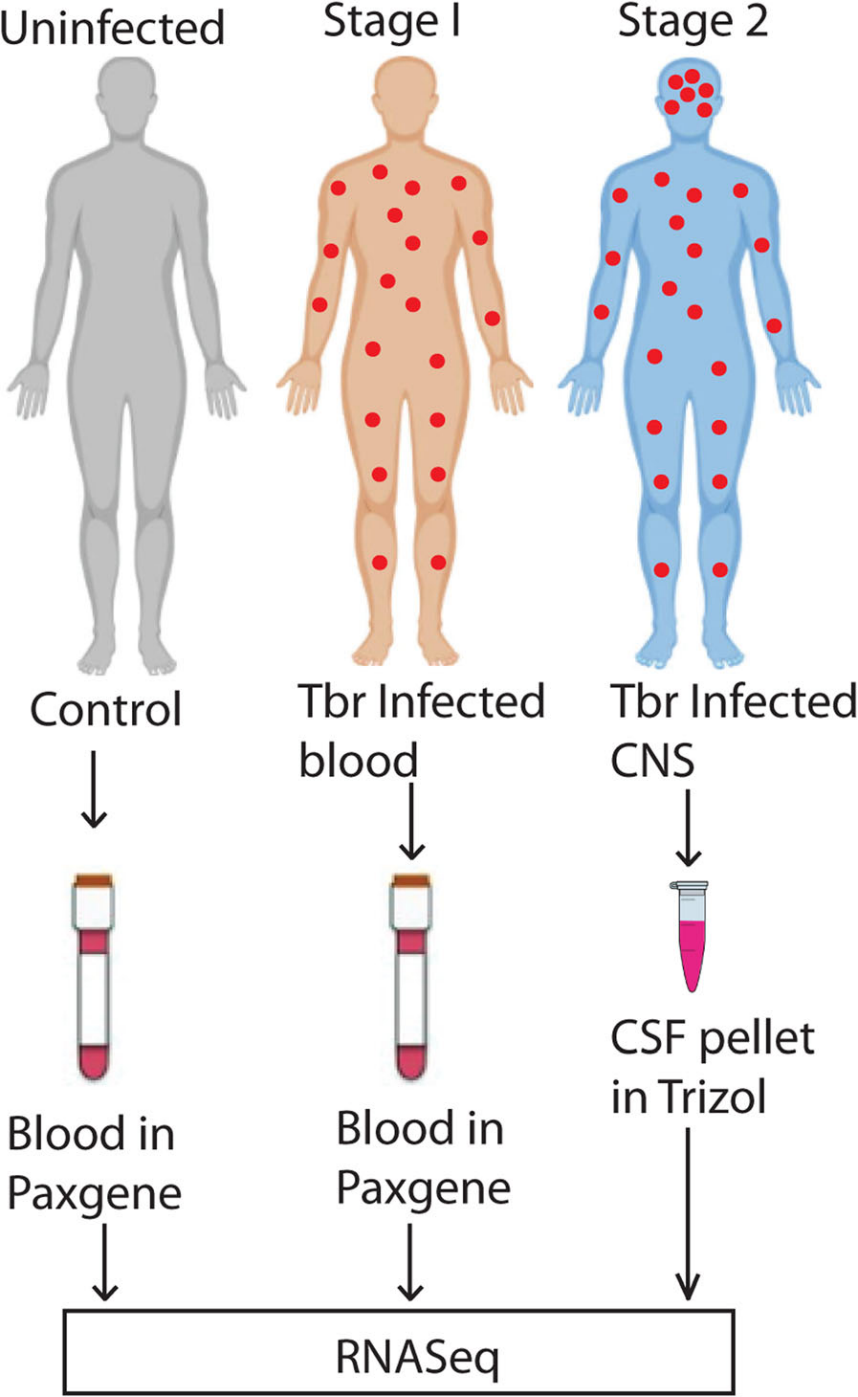
Study design schematic. The flow-chart shows how the sampling was carried out. Individuals in the first stage with parasites (red dots) in blood and the uninfected (normal) had peripheral blood in paxgene tubes. For individuals in the second stage (red dots in the head) CSF containing parasites was centrifuged and the cells were resuspended in trizol. RNA was extracted and sequenced on the illumina platform

The subjects for the study were recruited at Lwala hospital, Kaberamaido district, Eastern Uganda. The samples collected consisted of blood and cerebral spinal fluid (CSF) obtained from patients, with microscopic diagnosis of *T. b rhodesiense* parasites in the blood (early stage) and/or CSF (late stage). The confirmation of *T. b rhodesiense* parasites in the samples was carried out by species specific PCR of the serum resistance associated (SRA) gene; details of the infection characteristics can be found in Mulindwa et al. [17]. As controls, blood samples were obtained from uninfected healthy individuals from the same focus (Fig. 1). Nine subjects were used for this analysis; however, it is worth noting that one individual had both the blood and CSF samples analysed (Table 1). These nine subjects were selected on the basis of having good RNA yield and high parasitaemia as previously described [17]. The study subjects were from the same Kumam speaking Luo ethnic group and consisted of 4 females and 5 males with age ranging from 6 to 35 years. All the cases showed presence of *T. b rhodesiense* parasites with higher parasitaemia observed in blood (mean parasite count/ml, 3.2 × 10^7^) than the CSF (mean parasite count/ml, 3 × 10^5^). The RNA was extracted from Paxgene blood, and rRNA and haemoglobin mRNA were depleted prior to cDNA library preparation. For CSF, RNA was extracted from the frozen cellular fractions in Trizol and rRNA depleted. For comparison purposes, we tried placing CSF in Paxgene tubes but did not succeed in recovering RNA. All samples were reverse transcribed and sequenced. We previously analysed the transcriptomes of the trypanosomes in the blood and CSF samples [17]. Here, we have studied the transcriptomes from the human host, comparing them with blood RNA from three uninfected controls, prepared as for the HAT patients. The reads were aligned to the Human reference genome build GRCH38 (Table 1) using TOPHAT [19]. The average number of mapped reads per sample was 104 ± 71SD million single end reads.

**Table 1:** Summary data on the samples collected and RNA sequencing output

Sample code	Sex	Age	Disease stage	Sample type	WBC (10e4)	Tryps (10e4)	RNA (μg)	Input reads	GRCH38 mapped	Mapped >1X	% reads aligned
HC57	F	35	II	Csf	38	13	0.2	98,270,230	69,285,928	69,285,928	70.5
HC60	F	17	II	Csf	120	8	0.7	96,648,788	90,492,957	74,616,029	93.6
HC71	M	20	II	Csf	na	50	0.5	73,865,867	56,272,142	29,457,308	76.2
HB73	M	30	I	Blood	na	4500	8.1	123,187,268	93,326,386	23,674,401	75.8
HB71	M	20	II	Blood	na	5100	8	161,694,848	140,878,954	47,536,097	87.1
HB80	F	23	I	Blood	na	2100	2.6	149,174,871	45,622,196	6,551,610	30.6
HB81	F	6	I	Blood	na	1100	3.0	117,399,517	87,010,317	34,355,813	74.1
Control_1	M	12	–	Blood	na	na	5.3	348,313,772	294,908,395	73,030,586	84.7
Control_2	M	22	–	Blood	na	na	4.7	119,677,407	100,002,617	82,853,862	83.6
Control_3	M	17	–	Blood	na	na	7	87,008,230	71,454,798	28,084,167	82.1

### Correlation of genome wide expression across samples

The mapped reads were normalized for sequencing depth and gene length to obtain Reads Per Kilobase per Million mapped reads (RPKM) values, which were used to analyse for sample sequence quality. The blood case and control samples had similar median values except for samples 81B, Control 2 and Control 3, whereas the CSF samples except for 60C also had similar median values (Additional file 1: Figure S1A). Analysis of distance between the samples using the Jensen-Shannon algorithm showed that the samples under the same category of CSF, blood Cases or Controls had the least divergence between them although highest distance was observed between the CSF and blood samples (Additional file 1: Figure S1B). There was an even dispersion of RPKM mean counts within each sample (Additional file 1: Figure S2) and similarly, pairwise comparison of sample RPKM values showed a higher correlation (R > 0.8) between transcripts of blood samples (Cases and controls) than between the blood and CSF samples (R < 0.7) (Additional file 1: Figure S3) although significant genes were observed in all samples (Additional file 1: Figure S4).

### Sample transcriptomes clustered by phenotype category

**Fig. 2:**
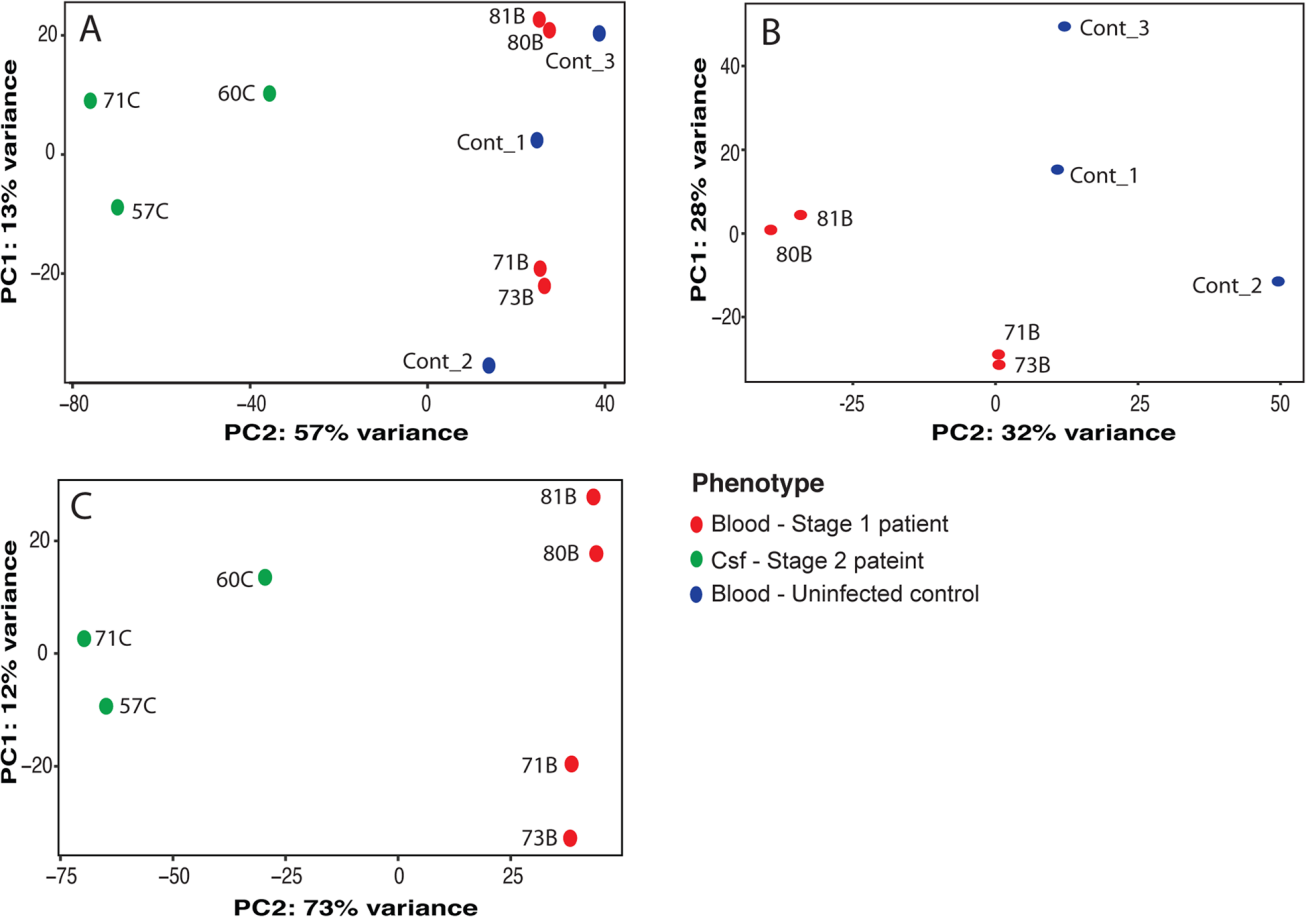
Sample stratification analysis. Principal component analysis (PCA) plot of the PC1 vs PC2 values for; **a** All samples, stage 1 (blood), stage 2 (CSF), and control samples, **b** comparison of stage 1 and control blood, **c** comparison of stage 1 blood and stage 2 CSF samples

To determine the differences in gene expression in the circulating blood and CSF of patients that result from *T. b rhodesiense* infection, we used DESeq2 to analyse the gene read count data output from Cuffdiff [21]. Using the DESeq2 data normalized by the variance stabilizing transformation (VST) algorithm, we determined the sample stratification by principal component analysis (Fig. 2). We observed that the CSF and blood transcriptomes formed distinct clusters with over 50% PC2 variance between them (Fig. 2a). However, there was less variation (30%) observed between the blood cases and controls (Fig. 2b, Additional file 1: Figure S5A), with approximately 838 genes differentially expressed (padj < 0.05) between cases and controls (Additional file 1: Figure S7Ai). A somewhat extreme variation (> 70%) was observed between the stage 1 (blood) and stage 2 (CSF) individual transcriptomes (Fig. 2c, Additional file 1: Figure S5B), with approximately 4994 genes differentially expressed between them (Additional file 1: Figure S7Aii). The downstream differential gene expression analysis was carried out between the Stage 1 and uninfected controls (Fig. 2b) and the Stage 2 [CSF] and Stage 1 [blood] (Fig. 2c).

### Enrichment of innate immune response transcripts during the early hemolymphatic stage of infection

**Fig. 3:**
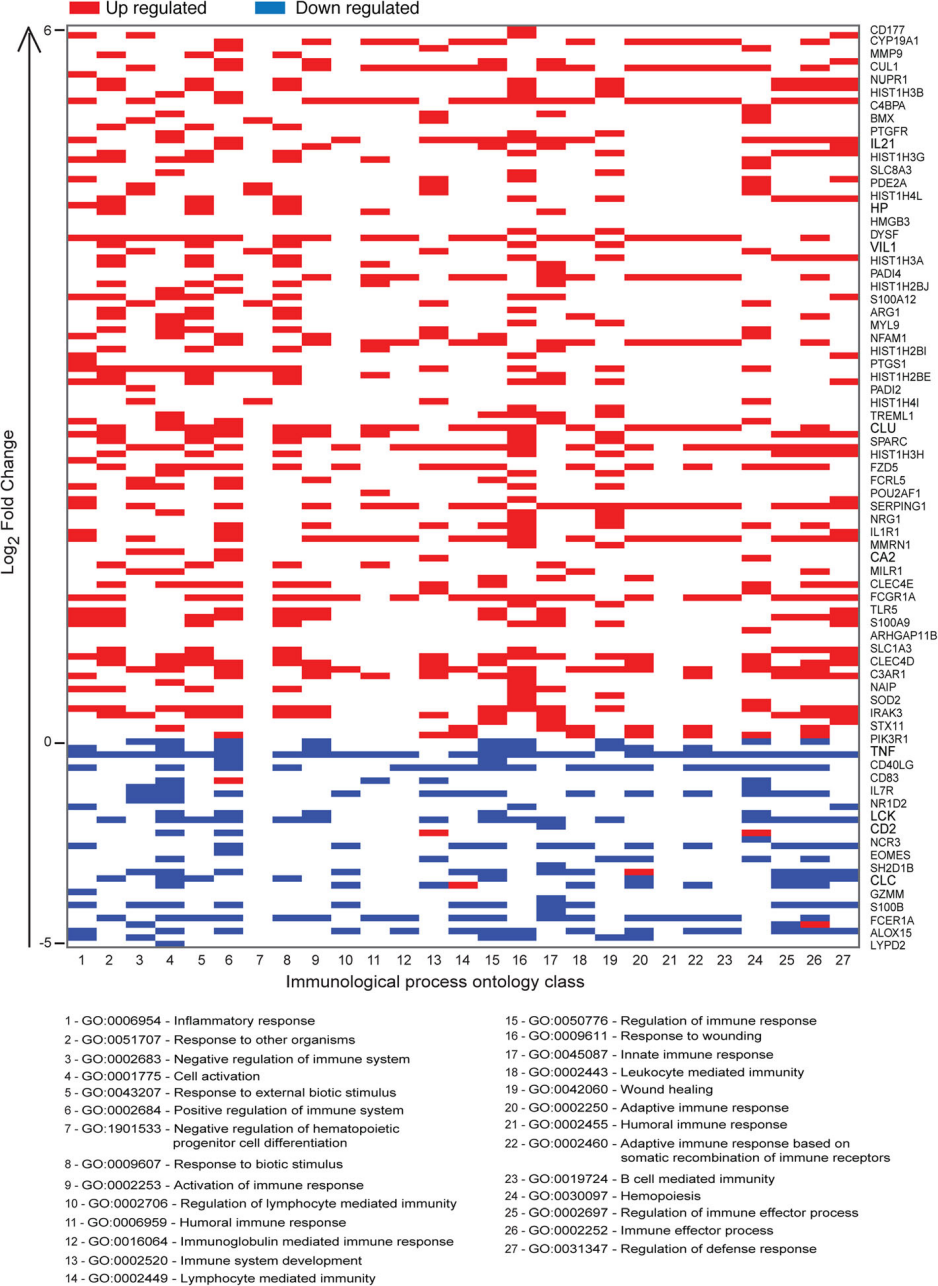
Pathway enrichment analysis using Toppcluster. The heatmap shows the over-expressed immune response genes that were enriched (*p* < 10E10) in the samples from infected patients relative to control individuals

To determine the differentially expressed transcripts resulting from *T. b rhodesiense* infection in the early hemolymphatic stage, we compared the blood transcriptomes from the cases and control individuals but excluding sample 80B due to its low number of reads (Table 1). We identified genes which are differentially expressed with an absolute log2 fold change (Log2FC > 1.0) using the apeglm estimator [26], which corrects for effective size shrinkage by removing noise and preserving large differences (Additional file 1: Figure S6 A). From this dataset, we extracted significantly differentially expressed genes (adjusted *p* value, padj < 0.05), and annotated them using the Ensembl database (Additional file 2: Table S1). This identified 839 significant differentially expressed genes (DEGs) of which 55% (462/839) coded for proteins (Additional file 1: Figure S7 Ai). Of these protein-coding genes, 33% (154/462) were down regulated (log2 fold change < − 1) whereas 67% (308/462) were up regulated (log2 fold change (log2FC) > 1) relative to the healthy controls. The DESeq2 rlog transformation of read counts (Additional file 1: Figure S6 B) was used to present these significant coding sequence (CDS) genes in a clustering heatmap using Euclidean distance correlation with complete linkage (Additional file 1: Figure S8). In order to determine which biological processes are most affected by *T. b. rhodesiense* infection, the CDS gene list was analysed for functional enrichment in cellular biological process genes using ToppCluster [22] and selected for genes with a Bonferroni corrected *p* value cut-off *p* < 0.05. For this we observed enrichment of 30% (139/462) genes annotated with immune response mechanisms (Fig. 3, Additional file 1: Figure S7 Bi, Additional file 2: Table S2). We observed up-regulation (log2FC > 2.0) of the classical complement pathway genes (C1QA, C1QB, C1QC, C3Ar1, C4BPA, CR1) which initiate antigen-antibody binding and formation of the C3 convertase [27]. Furthermore, high levels of HLA-DRB5 (log2FC 3.0), involved in presenting peptides from extracellular proteins and the immunoglobulin heavy chain variable transcripts (IGHVs, log2FC 3.0–6.0) were observed; these IGHVs are also involved in antigen presentation [28]. Looking at the cytokine levels, there was observed up regulation of pro-inflammatory TNF-α induced proteins (TNFAIP6 [log2FC 2.6], TNFAIP8 [log2FC 5.3]) involved in systemic inflammation; and also, elevation of interleukins IL21 (Log2FC 3.7) and IL1 receptor (Log2FC 2.0) that respond to infection. In addition, there was up regulation of Haptoglobin (HP, log2FC 3.1), which binds to haemoglobin to form the HpHb complex which is involved in innate immunity against Trypanosoma infection [29]. In addition up-regulated surface markers included CD163, a marker for macrophages and scavenger receptor for the haemoglobin-haptoglobin complex [30, 31], and CD177, involved in neutrophil activation [32]. These results implicate an innate immune response pathway role during the hemolymphatic stage. Within the up-regulated genes, we observed a significant enrichment within the KEGG pathways of the Systemic lupus erythematosus (SLE) pathway (FDR 2.3E-31) (Additional file 1: Figure S9). This possibly implied that the pathological immune response mechanism observed during *T.b rhodesiense* infection could be related to that in SLE [33]. A network analysis of the up-regulated genes indeed showed that they were involved in innate immune response pathways (Fig. 5a). The key gene hubs with multiple nodes in the network were Platelet factor 4, PF4, and thrombospondin 1, THBS1, which trigger innate response and cellular motility/adhesion mechanisms respectively [34, 35].

### Anti-inflammation and neuro-activation during second stage CNS infection

**Fig. 4:**
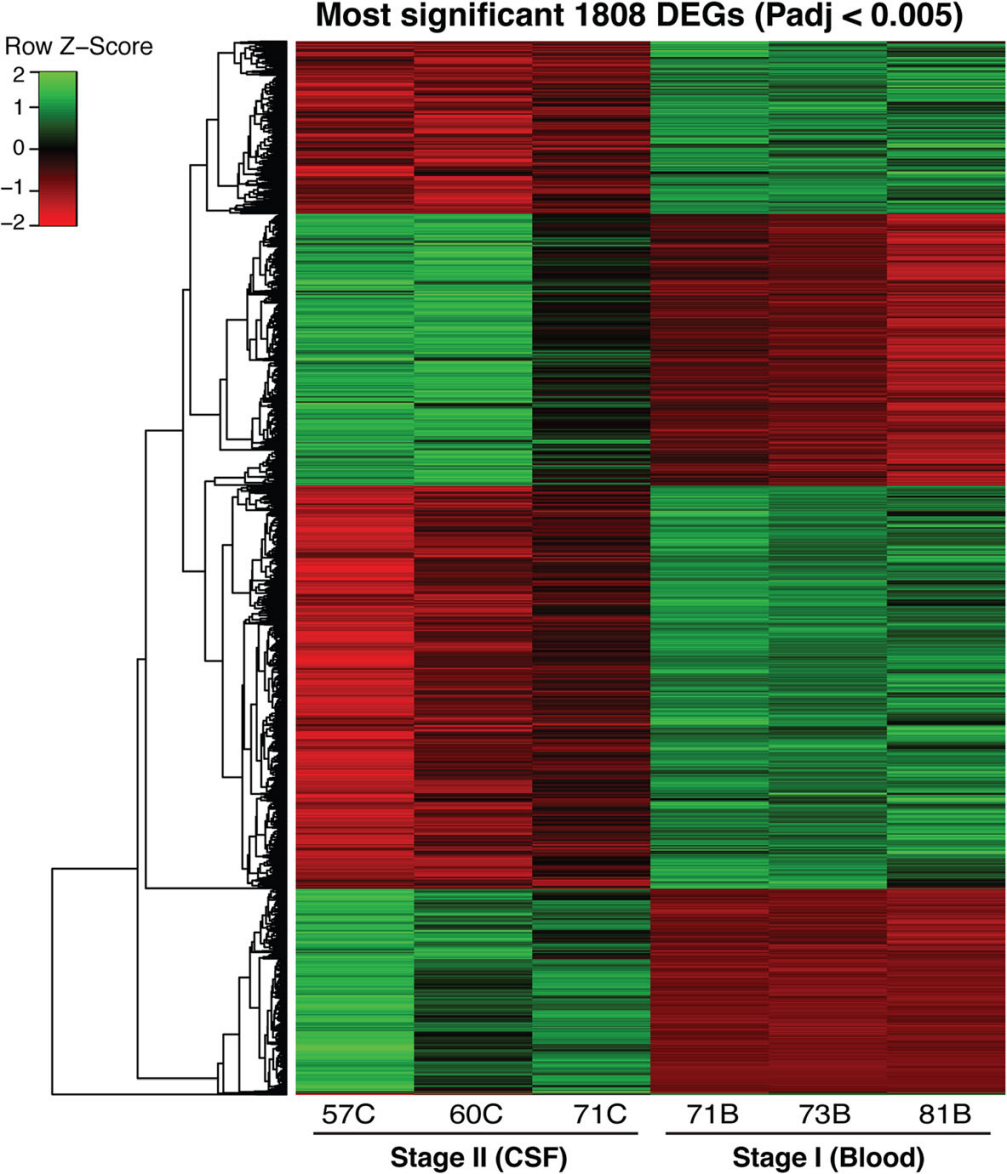
A clustering heat map (Euclidean distance correlation with complete linkage) showing the most significant differentially expressed genes (padj < 0.005) between the samples of stage II CSF and Stage I blood transcriptomes (1808 genes). The green intensity shows increased gene expression and the red indicates decreased gene expression

There is migration of circulating activated lymphocytes from the venous blood across the blood brain barrier or choroid plexus into the cerebral spinal fluid [36] . Therefore, to determine the genes which are differentially expressed in the blood and CSF lymphocytes during active *T. b rhodesiense* infection, and possibly identify mechanisms that distinguish the early and late stages of the disease, we compared the stage 1 blood (Table 1, HB73, HB71, HB81) and stage 2 CSF (Table 1, HC57, HC60, HC71) transcriptomes (Fig. 2c, Additional file 2: Table S3). CSF could not be obtained from uninfected people for ethical reasons; we chose to compare with the stage I, rather than stage II blood because we wanted to identify candidate biomarkers in blood that could be used to diagnose CSF infection. Four thousand two hundred thirty-four genes were differentially expressed at padj < 0.05 (Additional file 2: Table S3, and Fig. 4 which shows 1808 genes at padj < 0.005). Of these 52% (2232/4234) were up regulated (log2FC > 1) and 48% (2002/4234) down regulated (log2FC < − 1). When compared to the differential expression for the stage 1 blood samples versus control, there were over nine times more CDS significant genes (padj < 0.05) in the stage 2 (CSF) than stage 1 (blood) samples (Additional file 1: Figure S7A). Functional analysis of these DEGs showed enrichment for genes mainly in the biological processes of cellular organization, morphogenesis, motility and signalling (Additional file 1: Figure S7B.ii, Additional file 2: Table S4). In order to determine which pathways were enriched for in the up-regulated genes, we probed the innate immune response database [24]. We observed high enrichment (pvalue < 8.0E-4) of gene clusters involved in the ‘reactome’ pathways, that is, neuronal system, retinoid metabolism, diseases associated with visual transduction, visual photo-transduction and neurotransmitter receptor binding (Additional file 1: Figure S10). Genes within these clusters including ADCY2 and AKAP have been associated with bipolar disorder and schizophrenia [37, 38]. Furthermore genes associated with gamma-aminobutyric acid (GABRA2, GABRB) have been implicated in sleep disorders [39] including ApoB [40]. IL-10 which has been previously observed up-regulated in the CSF of *T. b. rhodesiense* patients [41, 42] was indeed more expressed in the CSF (log2FC 3.1) in addition to the other cytokines including IL12, IL17RD, IL20RA, IL21R, IL36B, IL32.

**Fig. 5:**
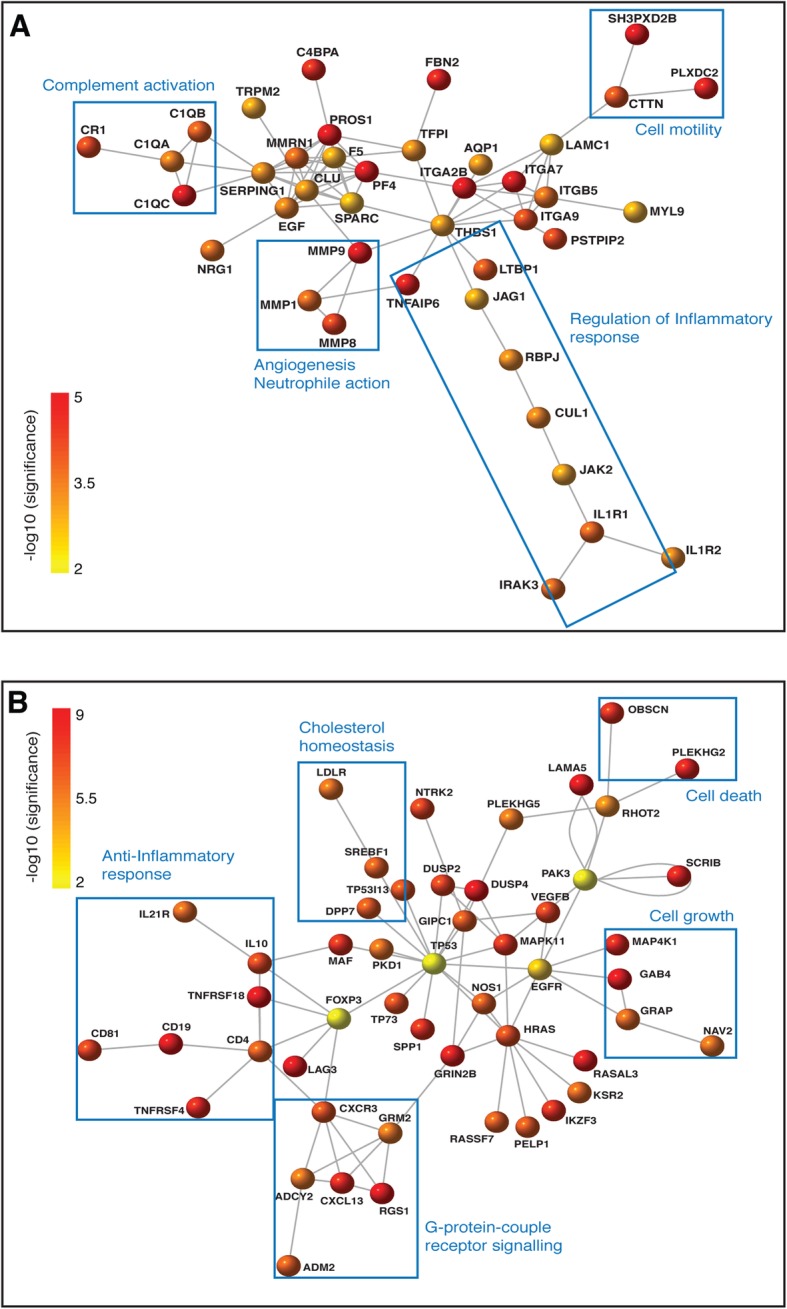
Pathway network analysis of the genes that are over-expressed (padj < 0.05) in the blood cases relative to controls (**a**) and in stage 1 blood vs CSF (**b**). The gene functional interactions network was used with high confidence scores > = 700. The nodes/genes were color-coded according to the -log10 (significance) with the most significant nodes/genes in the darker red

A network analysis of the up-regulated genes revealed a number of core signalling molecules and transcription factors involved in brain function (Fig. 5b). A key up-regulated factor in the network was FOXP3, which plays a fundamental role in the development and function of regulatory T cells (Treg, FOXP3^+^CD4^+^) and cellular proliferation and migration [43–45]. The CD4 receptor was up regulated (log2 FC 2.7) in addition to IL10 (log2 FC 3.1) a key anti-inflammatory cytokine linked to CD4+ T helper cells that interacts with MHC class II molecules that are generated from extra cellular pathogens [46], which in this case would be the trypanosomes in the CSF. The presence of FOXP3 and CD4 is an indication of elevated Tregs which is a result of inflamed central nervous system [47]. Furthermore, up-regulation of chemokine receptor CXCR3 (Log2FC 4.1) was indicative of CNS disease [48].

### Peripheral blood signatures for CNS infection

**Fig. 6:**
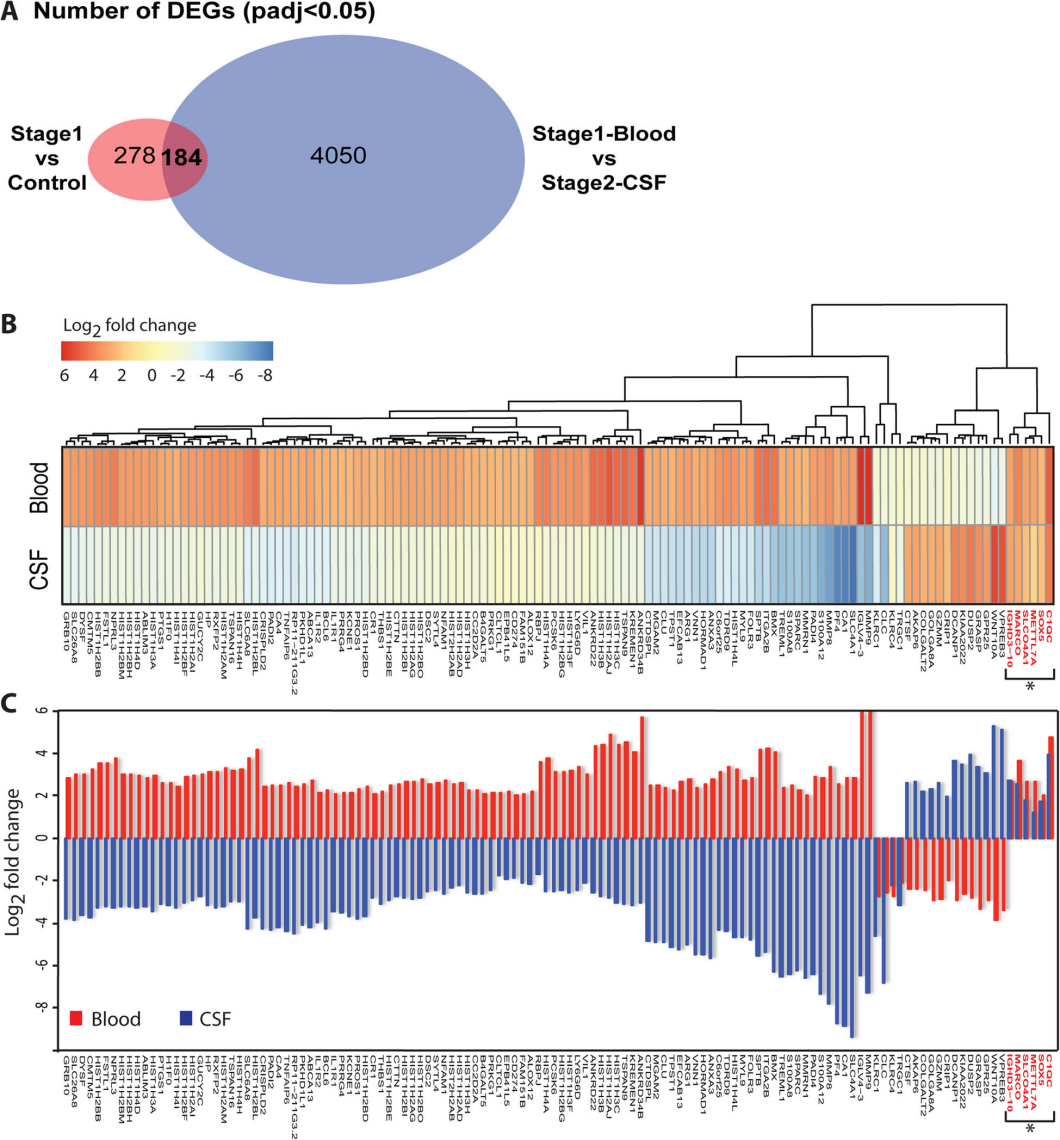
Comparison of differentially expressed genes (padj < 0.05) from stage 1 (Stage 1 vs controls) and Stage 2 (Stage 2 CSF vs Stage 1 blood) transcriptomes. **a** Venn diagram highlighting the number of genes that intersect the DEGs of stage 1 (Cases vs controls) and stage 2 (Stage 1 blood vs Stage II CSF). **b** Hierarchical clustering heatmap of the DEGs that are shared between the stage 1 and stage 2 transcriptomes, **c** Bar plot of the log2 fold change of the DEGs that are shared between the stage 1 and stage 2. *Shows the genes that have increased expression in both blood and CSF

We next looked at the genes that intersect between the DEGs for stage 1 and stage 2 individuals in order to identify genes co-expressed in both the blood and CSF (Fig. 6a). We identified a total of 184 genes that are significantly differentially expressed (padj < 0.05) both in blood (Stage 1 vs Controls) and CSF (Stage 2 [CSF] vs Stage 1[Blood]) transcriptomes (Additional file 2: Table S5). Over 90% of these genes increased in blood and decreased in CSF, suggesting an antagonistic role played by them during the course of infection. However following hierarchical clustering, we identified 6 genes (C1QC, SOX5, METTL7A, SLCO4A1, MARCO and IGHD3–10), which were increased in both the blood and CSF of stage 2 patients (Fig. 6b). Furthermore, C1QC, MARCO and IGHD3–10 were increased more than 5-fold in the blood of stage 1 patients (Fig. 6c). If the corresponding polypeptides are similarly increased, they might in future be considered as possible diagnostic markers for CNS invasion. C1QC forms part of the complement component 1q, which constitutes the innate immune system [49]; MARCO is a scavenger receptor found on macrophages and involved in phagocytosis of pathogens [50]; IGHD3–10 Immunoglobulin heavy chain diversity antigen receptors expressed by B cells and are a major component of the adaptive immune response [51].

## Discussion

In this study we present transcriptome data from both the peripheral blood and cerebral spinal fluids of individuals diagnosed with trypanosome infections in order to broadly identify disease signatures of infection.

The individual samples collected from the early and late stage of the disease showed a defined stratification based on their phenotype. There was lower variation between the transcriptomes from blood (38%, infected vs uninfected) than between the blood and CSF (70%, Infected). In both cases, we were sequencing mainly the transcriptomes of nucleated blood cells, but nevertheless, some other cells may have been present. For ethical reasons, we could not obtain CSF samples from healthy controls. Therefore, the fact that the samples originated from different body compartments is likely to be the major source of differential expression. The sample preparation procedure could also explain some variation observed between the CSF and blood samples: the CSF was centrifuged and the cell pellet resuspended in Trizol, whereas the blood samples were directly placed in Paxgene tubes, in which cells were lysed and RNA stabilized. However, we tried placing CSF in Paxgene tubes but did not yield RNA. An alternative consideration for this variation between the blood and CSF transcriptomes were the potential experimental covariates, sample structure and possible batch effects.

Our data showed an activation of innate immune response pathway genes in peripheral blood from patients with early stage disease. We observed over-expression of the classical complement pathway factors that stop at non-lytic C3 convertase on the trypanosome surface [52, 53]. There were high levels of immunoglobulin heavy chain variable transcripts (IGHVs), and HLA-DRB5 up-regulation could possibly be due to the priming by the numerous variable surface glycoproteins (VSGs) that maintain chronic infection by the circulating trypanosomes [54]. Increased expression of Th1 pro-inflammatory cytokines of TNF-α and IL-1 has been observed previously in *T. b rhodesiense* patients [41]. However elevated levels of IL21, which is produced by and regulates Natural Killer T-cells [55], would result in the observed down regulation of NK cell receptors (KLRC1, KLRC4, KLRB1) in addition to down regulation of a repertoire of T-cell receptors and hence reduced T cell activation.

For the late stage infection, which was demonstrated by the presence of parasites in the cerebral spinal fluid in the samples analysed [17], we observed an up-regulation of genes that are broadly involved in anti-inflammatory response. Consistent with previous cytokine measurements [41, 42], we observed elevation in anti-inflammatory cytokine mRNA levels including IL-10. This anti-inflammatory response could be attributed to the observed over expression of FOXP3 which is a key factor in development of regulatory T cells [43]. In addition FOXP3 is known to induce elevated expression of chemokines, CXCL13, CXCR3 and CXCR5; CXCL13 has indeed been observed in CSF of *T. brucei* infection [56, 57].

Other differences between blood and CSF could be attributable to the different body compartments - although CSF is not normally thought to contain any cells other than leucocytes. For example, the CSF samples showed higher levels of mRNAs (Log2 FC > 6.0) from neurotransmitter genes that have been implicated in promoting wakefulness such as histamine receptor (HRH3), dopamine (DRD2) [58], and gamma-aminobutyric acid receptors (GABR, Log2FC 5.0–7.0), which are implicated in sleep disorders [39]. These sleep disorders could be associated with the description of sleeping sickness patients as sleepy by day and restless by night [8]. There was observed elevation of transcripts involved in the G-protein coupled receptor pathway which are indicative of neuropathology as seen in other neuro-degenerative diseases [59].

To this date, the staging of HAT patients still relies on microscopic examination of CSF (obtained by lumbar puncture) for increased white blood cell count (>20cells/μl) and presence of trypanosomes [5]. The lumbar puncture method is quite painful and stressful to the patients that an alternative method would ensure compliance. Since blood is taken to determine the early hemolymphatic stage, the same blood sample could be used to determine the second CNS stage. For this we have identified mRNAs - C1QC, MARCO and IGHD3–10 – that have increased expression in both the early and late-stage patients.

## Conclusion

In this study, we have compared the transcriptomes of human cells of early stage and late stage HAT patient blood and cerebral spinal fluid. We have identified a number of transcripts that are involved in the signalling and production of cytokines and chemokines that have been detected in *T. b rhodesiense* patients hence determining the underlying mechanisms for the observed pathology. Our study has also identified potential biomarkers as signatures that could be explored in the dual detection of both early and late stage disease in the same blood sample. However, this study was under powered, with a limited sample size due to technical challenges in obtaining samples for dual host and parasite, and CSF transcriptome analysis. Therefore, we recommend validation of the identified biomarkers on a larger cohort of Rhodesiense sleeping sickness patient samples.

## Supplementary information

**Additional file 1: Figure S1.** A. Box plot of rpkm values (fragments per kilobase per million mapped reads) for all the samples. B. Analysis of the Jensen-Shannon (JS) distance heatmap showing the pairwise divergenge between samples. **Figure S2.** Scatter plot comparing the mean counts (rpkm) against the estimated dispersion for each of the samples. **Figure S3.** Scatter plot based on the pairwise log RPKM values between all the samples. **Figure S4.** Volcano plot highlighting the signifcant genes (red) in each of the samples. **Figure S5.** Heatmap of clustering matrix comparing A. blood cases and controls and B. cases and CSF. **Figure S6.** Comparison of data normalization algorithms. **Figure S7.** A. Pie charts representing the propostion of significant differentially expressed genes (DEGs) assigned to 4 main Ensembl annotation categories of CDS (genes coding for functional and structural proteins), RNA coding genes, Ribosomal protein coding genes, Pseudogenes and Novel genes. B. Biological function enrichment analysis of the DEGs in stage 1 (i) and stage 2 (ii). **Figure S8.** A clustering heat map (Euclidean distance correlation with complete linkage) showing the most significant differentially expressed genes (padj < 0.05) between the blood cases and control transciptomes (462 genes). **Figure S9.** Enrichment analysis for KEGG pathway genes.**Figure S10.** InnateDB output of significantly enriched pathways in the differentially expressed genes (padj<0.05) between blood cases and CSF samples.

**Additional file 2: Table S1.** Cases vs Controls DEGs. **Table S2.** Cases vs Controls - Immune response gene enrichment. **Table S3.** Cases vs CSF DEGs. **Table S4.** Cases vs CSF - Gene functional annotation. **Table S5.** DEGs in the blood and CSF during active infection.

## Data Availability

The raw data are available at the EMBL-EBI Array express, https://www.ebi.ac.uk/arrayexpress/, under accession numbers E-MTAB-5293 and E-MTAB-5294.

## Abbreviations

CSF: Cerebral spinal fluid
DEGs: Differentially expressed genes
HAT: Human African Trypanosomiasis
