# Supplementary material for: Blood signatures for second stage human African trypanosomiasis: a transcriptomic approach
Source: BMC Med Genomics. 2020 Jan 30;13:14. doi: 10.1186/s12920-020-0666-5 (PMC6993467; doi:10.1186/s12920-020-0666-5)
Supplement: Supplementary file 1 — Additional file 1: Figure S1. A. Box plot of rpkm values (fragments per kilobase per million mapped reads) for all the samples. B. Analysis of the Jensen-Shannon (JS) distance heatmap showing the pairwise divergenge between samples. Figure S2. Scatter plot comparing the mean counts (rpkm) against the estimated dispersion for each of the samples. Figure S3. Scatter plot based on the pairwise log RPKM values between all the samples. Figure S4. Volcano plot highlighting the signifcant genes (red) in each of the samples. Figure S5. Heatmap of clustering matrix comparing A. blood cases and controls and B. cases and CSF. Figure S6. Comparison of data normalization algorithms. Figure S7. A. Pie charts representing the propostion of significant differentially expressed genes (DEGs) assigned to 4 main Ensembl annotation categories of CDS (genes coding for functional and structural proteins), RNA coding genes, Ribosomal protein coding genes, Pseudogenes and Novel genes. B. Biological function enrichment analysis of the DEGs in stage 1 (i) and stage 2 (ii). Figure S8. A clustering heat map (Euclidean distance correlation with complete linkage) showing the most significant differentially expressed genes (padj < 0.05) between the blood cases and control transciptomes (462 genes). Figure S9. Enrichment analysis for KEGG pathway genes.Figure S10. InnateDB output of significantly enriched pathways in the differentially expressed genes (padj<0.05) between blood cases and CSF samples. [file 12920_2020_666_MOESM1_ESM.pdf]

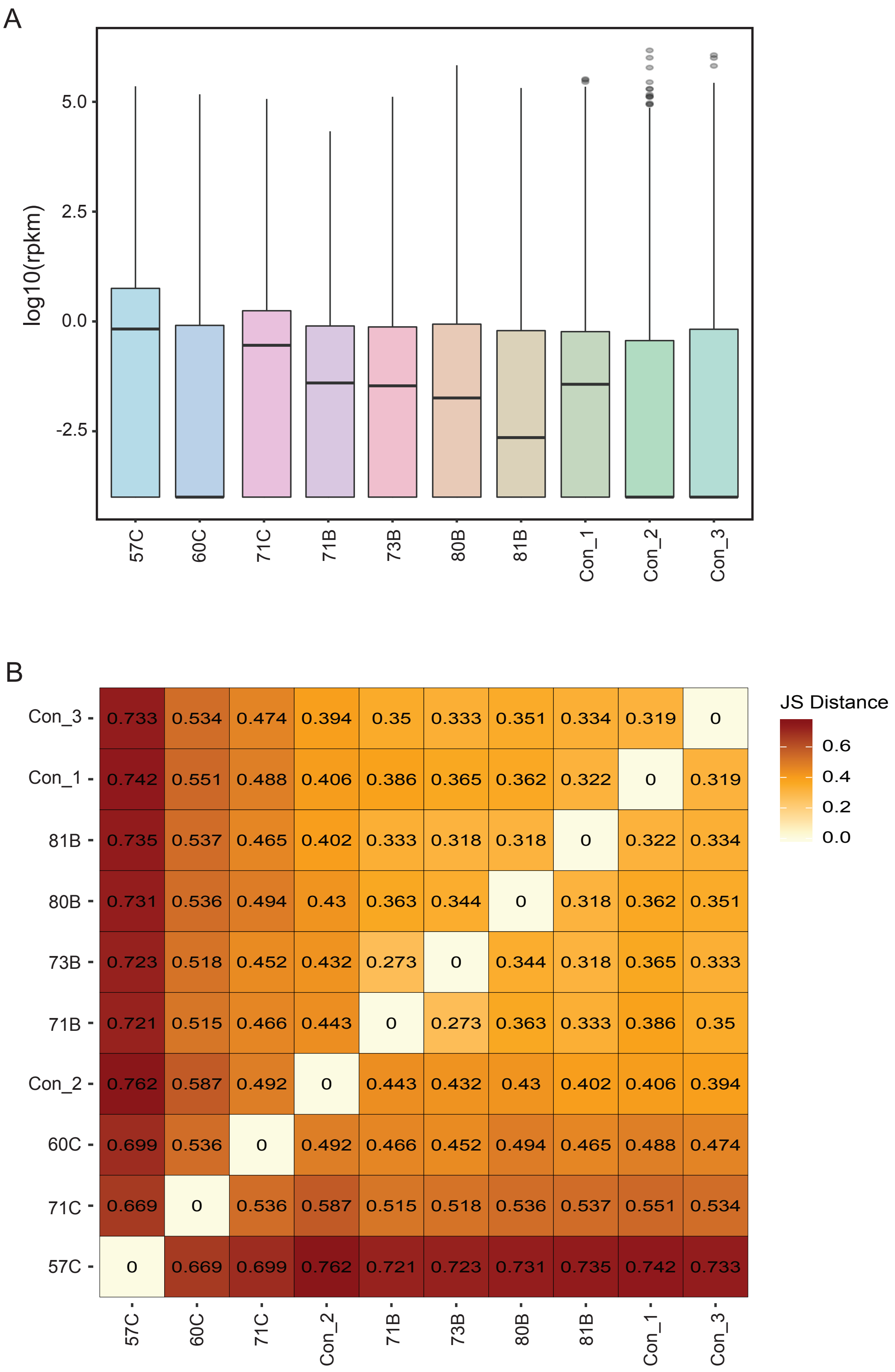

Figure S1 A. Box plot of rpkm values (fragments per kilobase per million mapped reads) for all the samples. The CSF samples had similar median values except for 60C, the blood case samples also had similar median values except 81B and control sample 1 had a median similar to the cases however control 2 and 3 were divergent. B. Analysis of the Jensen-Shannon (JS) distance heatmap showing the pairwise divergenge between samples. The higher the JS distance, the higher the sample similarity; samples from the same category CSF, Cases and Controls had least divergence between themselves.

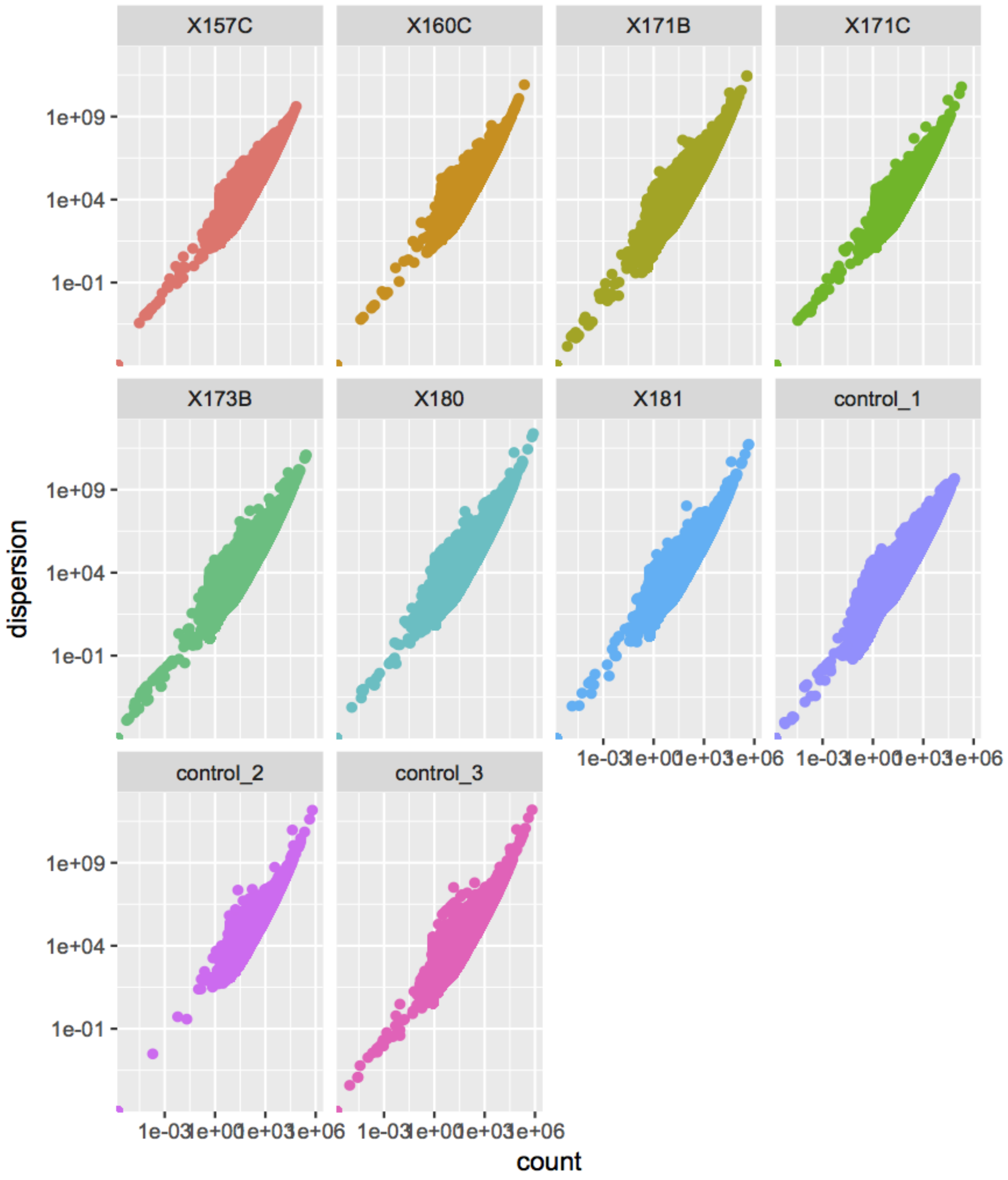

Figure S2. Scatter plot comparing the mean counts (rpkm) against the estimated dispersion for each of the samples. There was observed similar dispersion in all the samples and with minimal variability.

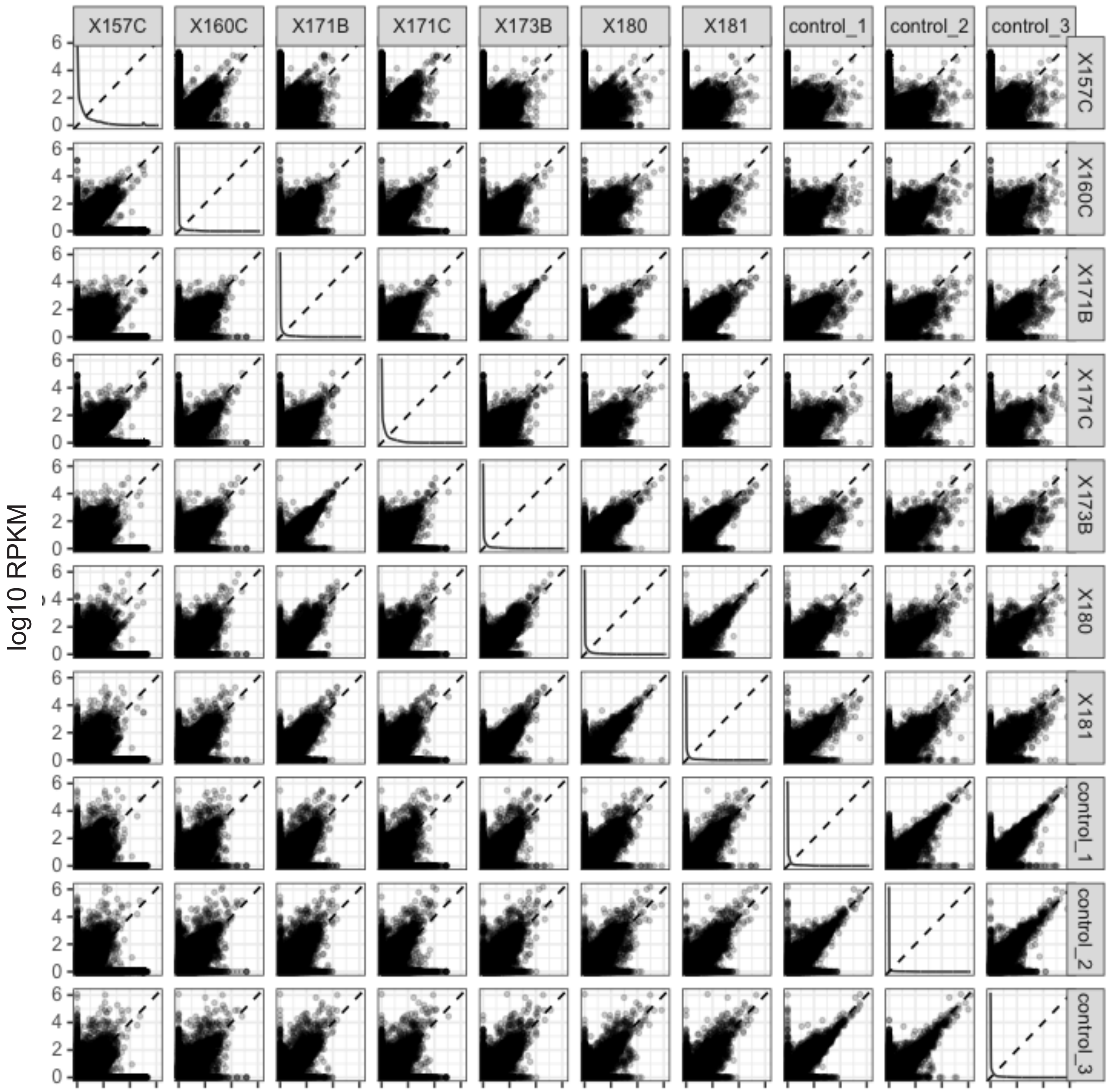

Figure S3. Scatter plot based on the pairwise log RPKM values between all the samples. The was a high correlation (>0.8) between blood samples (Cases and Controls) and lower correlation (<0.7) between the blood and CSF samples.

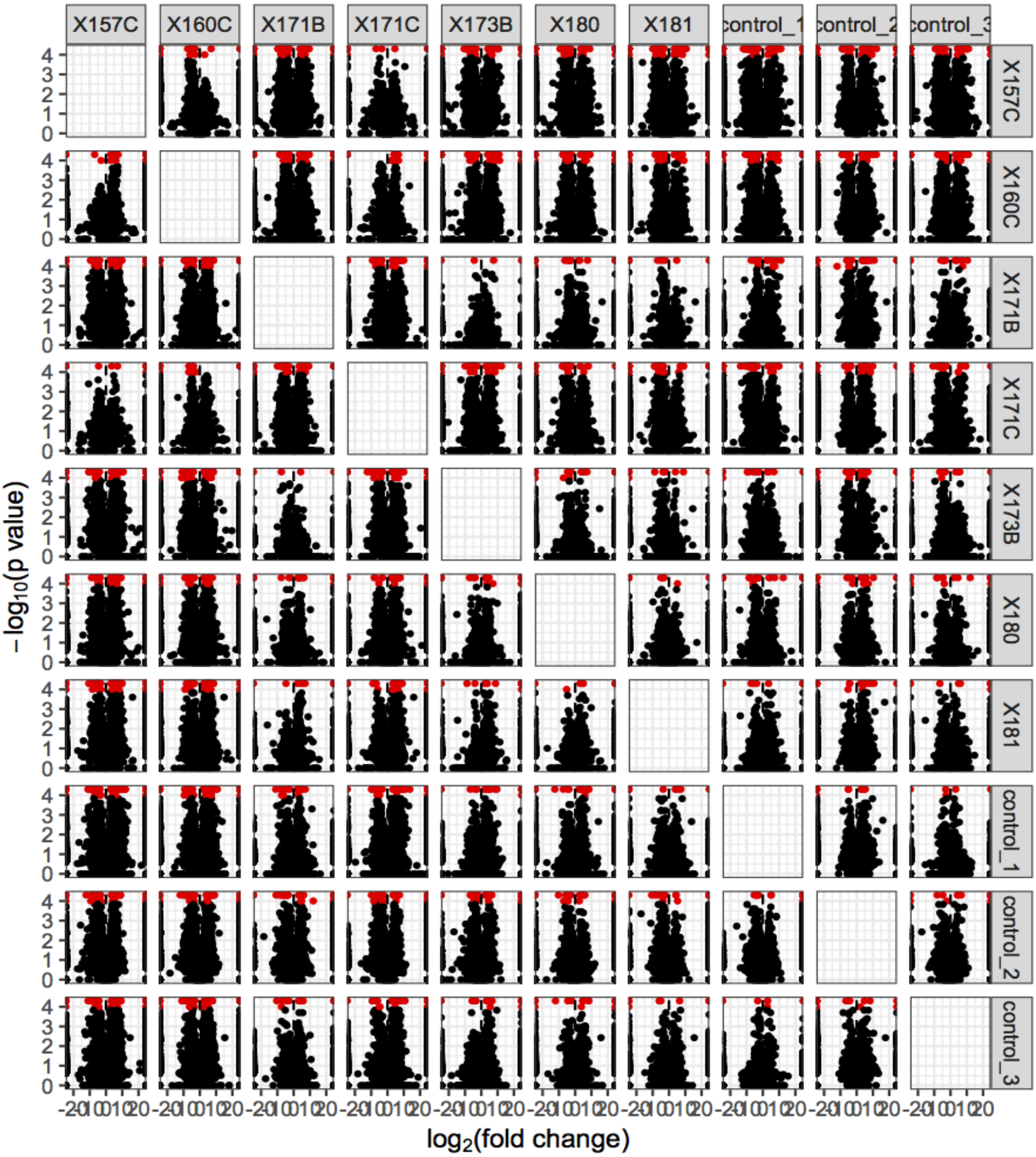

Figure S4. Volcano plot highlighting the signifcant genes (red) in each of the samples

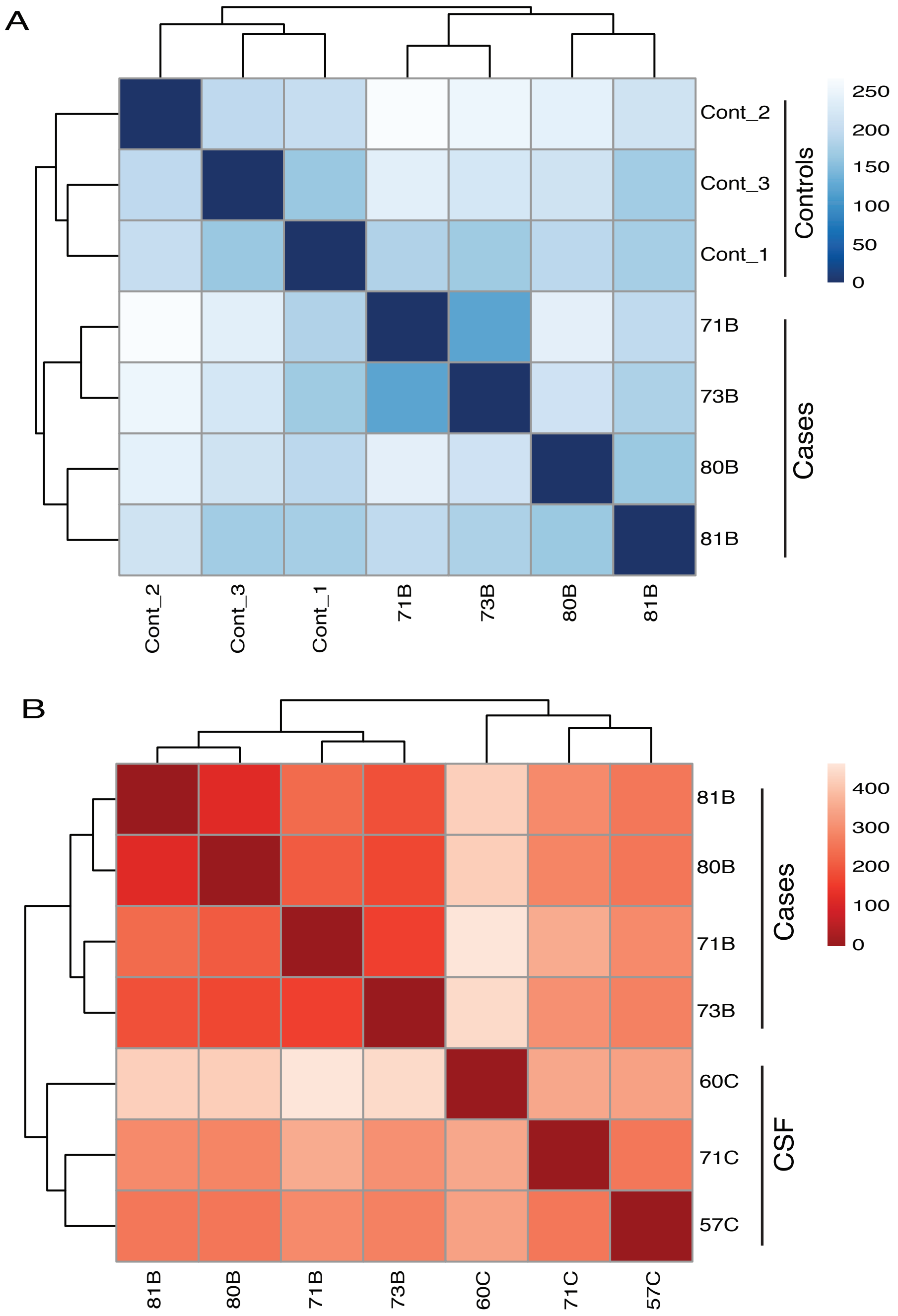

Figure S5. Heatmap of clustering matrix comparing A. blood cases and controls and B. cases and CSF

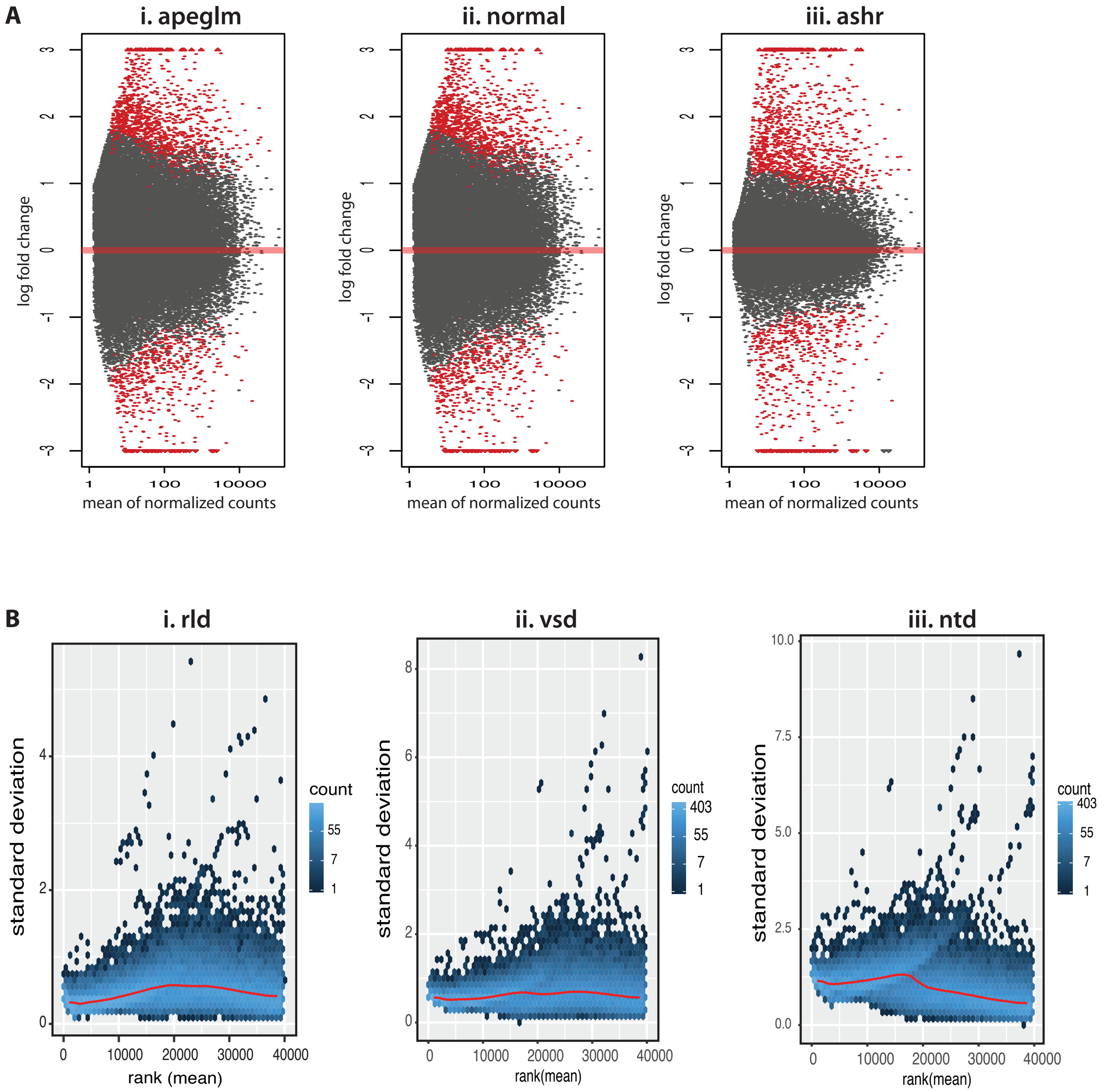

Figure S6. Comparison of data normalization algorithms **A**. The ratio intensity MA plot of the log fold change ratio verses mean of normalised count data of blood cases and controls comparing the DESeq2 shrinkage estimators of i. normal, ii. apeglm and iii. ashr. **B**. The plot of standard deviation of the transformed data (blood cases and controls) against the mean, comparing i. regularized log transformation (rld), ii. variance stabilizing transformation and iii. normal log transformation (ntd).

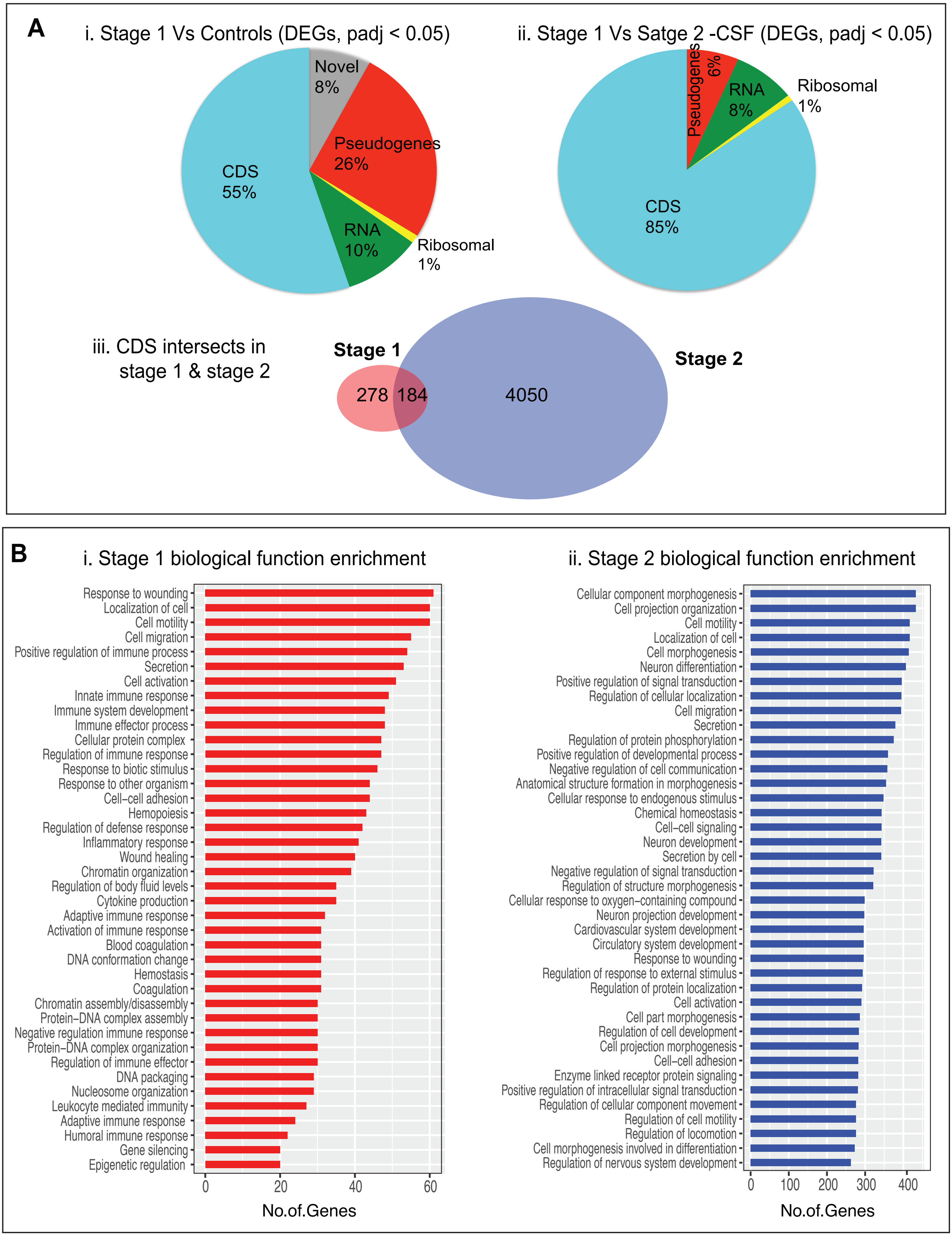

Figure S7. A. Pie charts representing the propostion of significant differentially expressed genes (DEGs) assigned to 4 main Ensembl annotation categories of CDS (genes coding for functional and structural proteins), RNA coding genes, Ribosomal protein coding genes, Pseudogenes and Novel genes. **Ai.** DEGs in blood cases vs controls, **Aii.** DEGs in blood cases vs CSF, **Aiii**. Intersection of DEGs in stage 1 & 2. B. Biological function enrichment analysis of the DEGs in stage 1 (i) and stage 2 (ii).

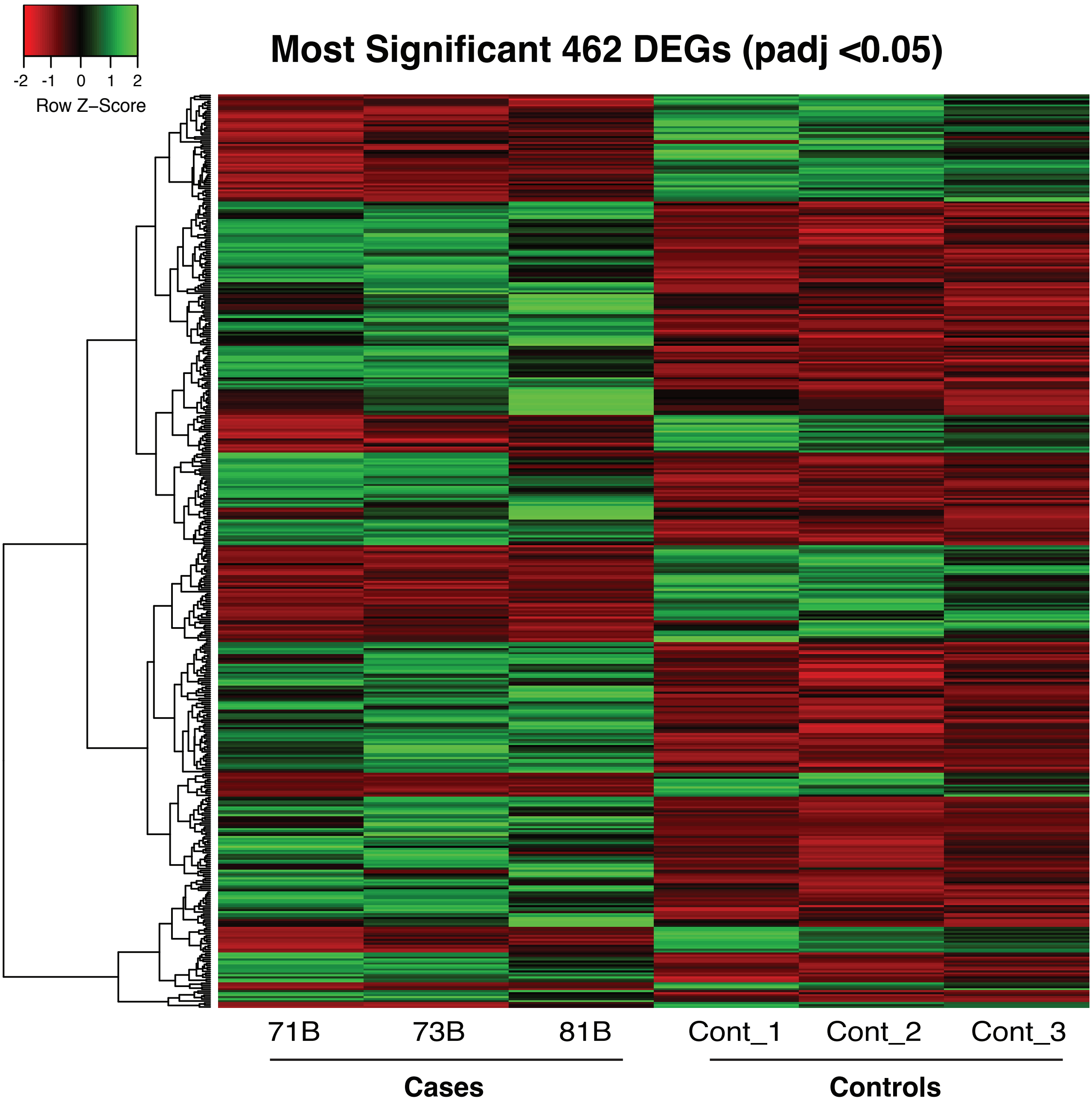

Figure S8. A clustering heat map (Euclidean distance correlation with complete linkage) showing the most significant differentially expressed genes (padj < 0.05) between the blood cases and control transciptomes (462 genes). The green intensity shows increased expressed and the red indicates decreased expression in *Tbrhodesiense* infected blood.

A

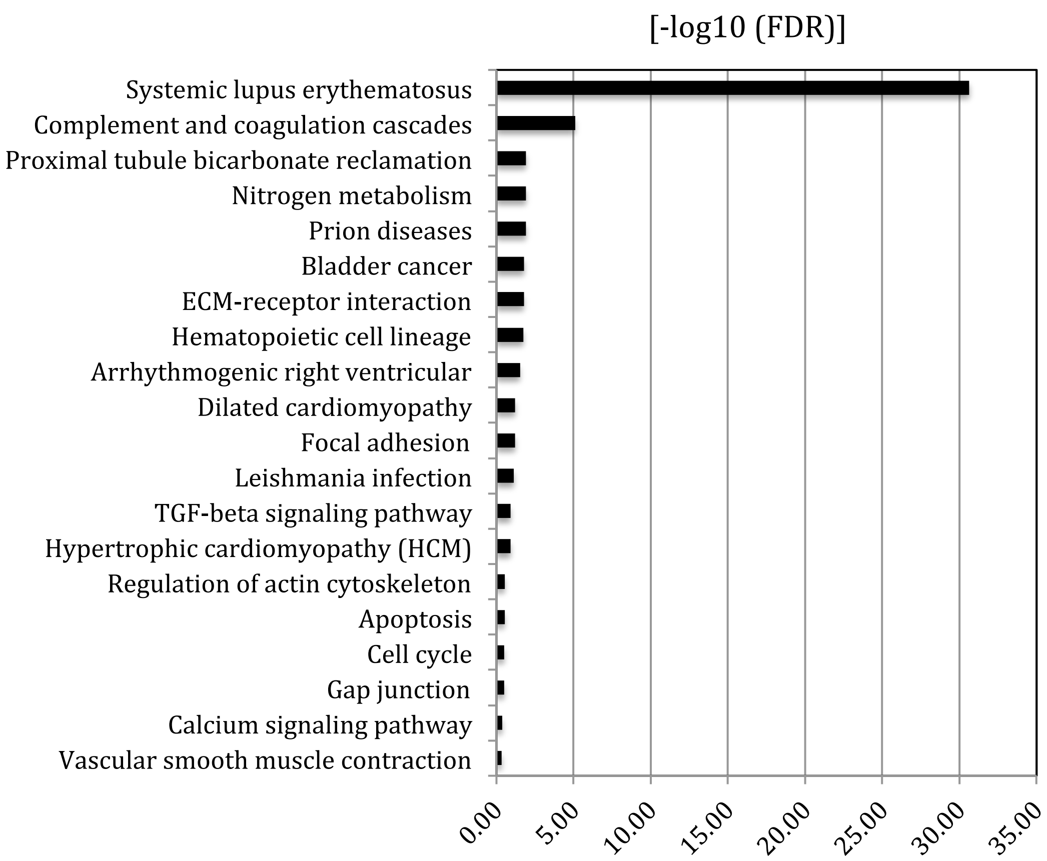

B

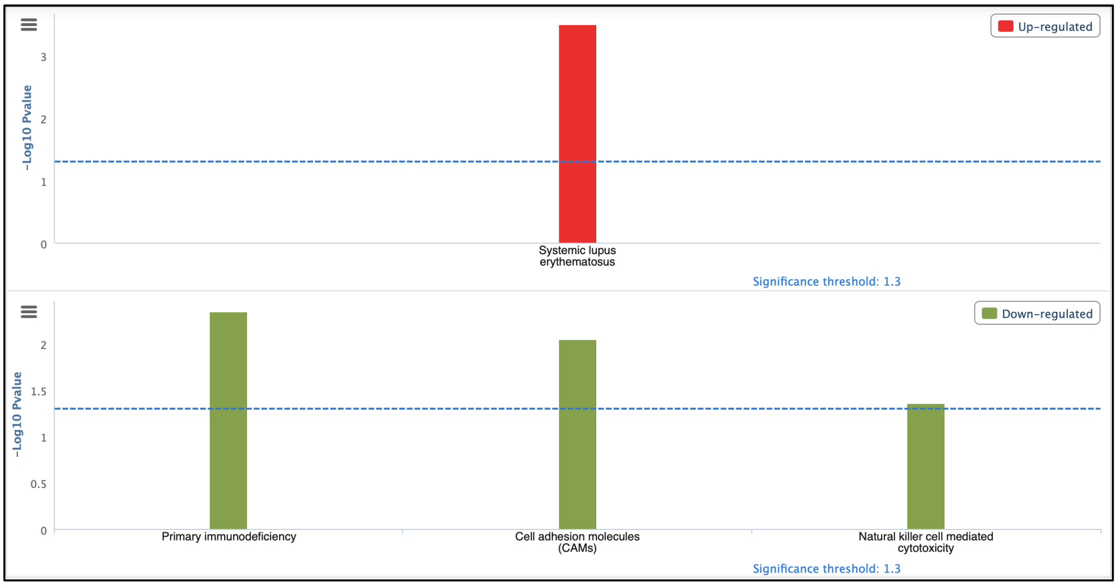

Figure S9. Enrichment analysis for KEGG pathway genes. **A.** XGR output of pathway vs false discovery rate values (FDR) **B.** InnateDB output of significant enrichment pathway

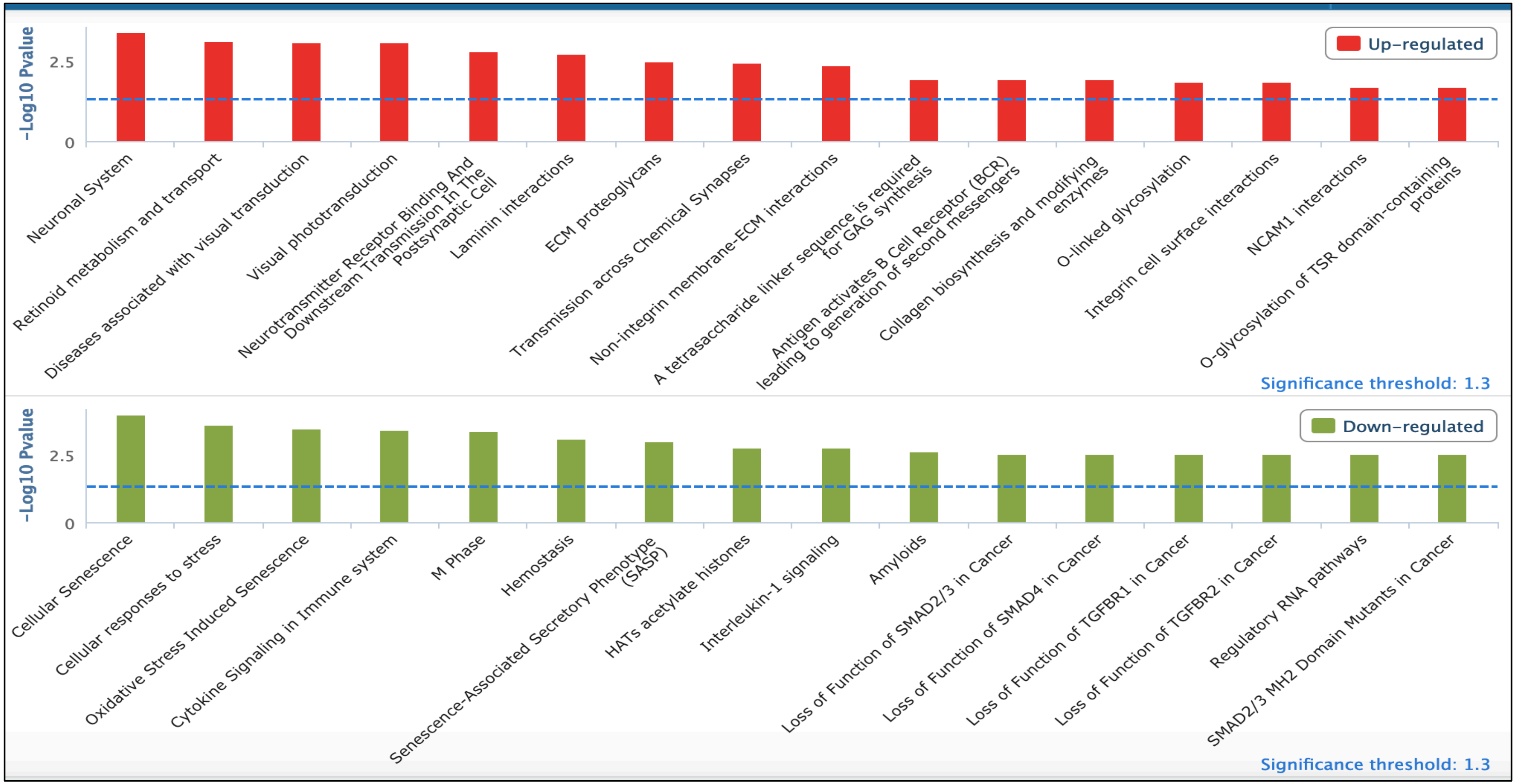

Figure S10. InnateDB output of significantly enriched pathways in the differentially expressed genes (padj<0.05) between blood cases and CSF samples.
